# Nurses as Health Visitors

**Published:** 1920-09-25

**Authors:** 


### Nurses as Health Visitors.

Out of a total of 1,879 health visitors employed under the Ministry of Health, 986, or more than half, are fully-trained nurses, and 1,360 (including many of the latter) are certificated midwives. In addition to these whole-time health-visiting nurses, there are 1,130 district nurses acting also as health visitors, but only giving part of their time, estimated as one-tenth, to this work. It is estimated that at least 2,000 health visitors are required to keep in touch with newly-born infants at the ratio of one to each 400 births, which has been lately established by the Local Government Board.

Health visiting is by no means, however, stereotyped, and it will be a matter for regret if it should become so. Ante-natal treatment has recently been withdrawn from its province and properly transferred to the midwives. This will set the health visitors free to devote their energies to children peculiarly in need of care. Nurse-children looked after by foster-mothers "for reward" have hitherto been under the inspection of "infant-protection visitors" appointed specially for this purpose. It is now proposed that health visitors shall supervise these instead—a very sensible alternative, likely to lead to economy. Other children who are to come within the health-visiting sphere are the motherless children of soldiers who fell in the war or are still serving. These children are provided for by the Special Grants Committee of the Ministry of Pensions, and are usually boarded out with relatives or foster-mothers at a rate of 12*s.* a week. It is now over a year since the new regulations instituting a special curriculum and certificate for health visitors were issued. The course of training laid down stipulated for one year's course in the case of trained nurses, and there should be a number of candidates now possessing the new certificate.

